# Raman Investigation of Cardiac Tissues with Sodium-Induced High Stiffness

**DOI:** 10.3390/molecules31030530

**Published:** 2026-02-03

**Authors:** Igor Artyukov, Gregory Arutyunov, Dmitrii Dragunov, Nikolay Melnik, Elena Perevedentseva, Vadim M. Mitrokhin, Anna Sokolova

**Affiliations:** 1 P. N. Lebedev Physical Institute, Russian Academy of Sciences, 53 Leninsky Prospect, 119991 Moscow, Russia; melniknn@lebedev.ru (N.M.); perevedencevaev@lebedev.ru (E.P.); 2 Department of Propaedeutics of Internal Medicine No. 1, Institute of Clinical Medicine, N.I. Pirogov Russian National Research Medical University, Ministry of Health of the Russian Federation, 117513 Moscow, Russia; arutyunov_gp@rsmu.ru (G.A.); dragunov_do@rsmu.ru (D.D.); mitrokhin_vm@rsmu.ru (V.M.M.); sokolova_av@rsmu.ru (A.S.)

**Keywords:** sodium overloading, sodium deposits, Raman spectroscopy, myocardial stiffness, glycosaminoglycan, collagen

## Abstract

This study investigates the molecular and mechanical effects of sodium accumulation in myocardial tissue using a combination of physiological measurements and Raman spectroscopy. Male Wistar rats were maintained on normal- and high-salt diets to induce differential sodium loading in cardiac tissue. Hemodynamic and mechanical analyses revealed increased myocardial stiffness and altered contractile parameters in the high-salt group. Raman microspectroscopy of myocardial sections demonstrated distinct spectral changes, particularly in regions corresponding to glycosaminoglycan (GAG), collagen, and its component, proline. Enhanced Raman signals near 1640 cm^−1^ in the Amide I range, 1246 cm^−1^ in the Amide III range, and in the 1030–1070 cm^−1^ range indicated structural modifications of the GAG–collagen complex and an increased contribution of proline-rich collagen, consistent with elevated tissue rigidity. These findings support the concept that sodium deposition in the myocardium alters its molecular architecture and mechanical properties through GAG-mediated binding and collagen remodeling. This study provides new insights into the biophysical mechanisms linking sodium homeostasis to myocardial stiffness and diastolic dysfunction.

## 1. Introduction

The impact of salt on the human body has traditionally been considered straightforward: higher salt intake increases the risk of hypertension and cardiovascular disease. However, recent advances in sodium (Na^+^) physiology have revealed that its role extends far beyond simple osmotic regulation. Emerging evidence indicates that sodium homeostasis is a far more complex biophysical process than previously understood, involving mechanisms that transcend the classical two-compartment (intracellular and extracellular) osmotic model [1]. Notably, sodium can be stored in osmotically inactive “reservoirs” within interstitial tissues, leading to the development of a “three-compartment model” of sodium balance [2,3,4]. In this model, sodium accumulation does not necessarily entail parallel water retention. Recent research suggests that excessive salt intake may promote sodium deposition in muscle tissues, thereby increasing the risk of cardiovascular complications, including fatal outcomes [5].

The concept of sodium storage in skin and muscle tissues was first proposed in the 1970s [6] and was later significantly advanced by Titze et al. [7,8,9], who provided important insights into this model. In particular, they highlighted the possibility of osmotically inactive sodium deposits bound to glycosaminoglycans (GAGs) within muscle tissue. Direct measurements of sodium content using ^23^Na MRI demonstrated clear evidence of sodium accumulation in skeletal [10,11] and cardiac muscles [12,13].

In our previous work, using synchrotron fluorescence X-ray microscopy [14,15], we showed that high sodium intake promotes sodium accumulation in the interstitial spaces of rat myocardium. Moreover, analysis of human myocardial samples revealed a strong correlation between sodium deposition and myocardial stiffness [16].

Alterations in stiffness—i.e., changes in the mechanical properties of the myocardium induced by excessive salt consumption—may serve as indicators of substantial physical and/or chemical tissue modifications. Such mechanical testing can be complemented by molecular-level analysis. In this study, we applied Raman spectroscopy to characterize myocardial samples that differed according to sodium intake and mechanical stiffness.

Raman scattering is an inelastic process in which incident photons exchange energy with vibrational levels of molecules or crystals, generating spectra that serve as molecular fingerprints of the material. This technique provides insights into chemical composition, molecular conformation, and the microenvironment of functional groups [17,18].

Raman spectroscopy requires minimal sample preparation, which enables studies of living or functioning biological specimens. Under controlled conditions (excitation wavelength, intensity, temperature, and medium), it is considered non-destructive. Consequently, it has become a powerful tool in biological research and is already widely applied to tissue studies [18,19,20]. Raman-based diagnostic methods, including in vivo applications, are actively being developed [19].

In particular, Raman scattering has been successfully used to study extracellular matrix components in bone, cartilage, cardiovascular, and neoplastic tissues [20], providing functional information that helps to distinguish between normal and pathological states. In cardiovascular research, Raman spectroscopy has been applied to vascular walls [21,22], particularly for detecting atherosclerotic changes. Moreover, it has been used to monitor collagen degradation in porcine aortic valves ex vivo, following enzymatic or cryopreservation treatment [23].

In this work, we investigate the effect of a high-salt diet on the molecular structure of rat myocardial tissue using Raman spectroscopy. By combining physicochemical analysis with structural assessment, we aim to identify molecular-level alterations that underlie the impact of excess sodium on myocardial mechanical properties, including hemodynamic function, stiffness, and elasticity. This approach provides new insight into the mechanisms linking sodium deposition to impaired cardiac performance.

## 2. Results

### 2.1. Hemodynamic Parameters and Myocardial Stiffness

**Figure 1:**
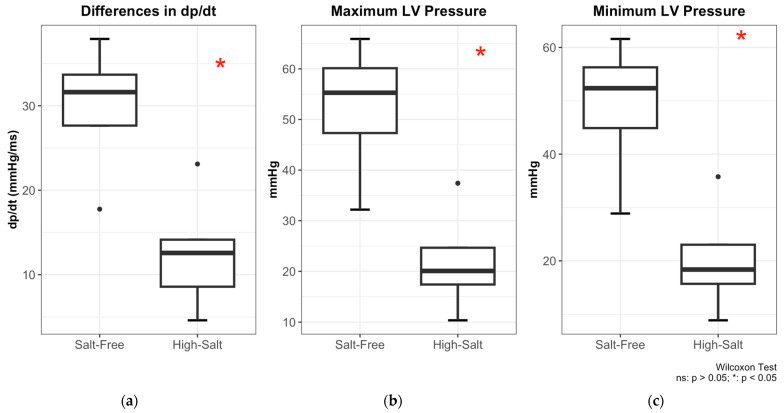
Hemodynamic effects of a HS diet in rats in reference to FS diet: comparison of (**a**) *dp*/*dt*; (**b**) maximum left ventricular (LV) pressure; and (**c**) minimum LV pressure. The central horizontal line within each box represents the median, while the lower and upper bounds of the box indicate the first (Q1) and third (Q3) quartiles, respectively. Whiskers extend to the most extreme values within 1.5× the interquartile range (IQR), and individual points denote outliers. Group comparisons were performed using the Wilcoxon rank-sum test; *p* < 0.05 indicates a statistically significant difference, while ns denotes a non-significant result (*p* > 0.05).

Figure 1 summarizes the key hemodynamic parameters of animals maintained on an experimental high-salt (“HS”) or control salt-free (“free-of-salt, “FS”) diet.

As shown in Figure 1a, the rate of blood pressure change (*dp*/*dt*) was significantly higher in the FS group. The mean *dp*/*dt* value in FS rats was 29.7 ± 8.5 mmHg/ms, nearly twice the value in HS rats (12.6 ± 6.9 mmHg/ms, *p* < 0.05). This reduction in *dp*/*dt* in the HS group indicates impaired left ventricular contractility under high-salt conditions.

Figure 1b shows that the maximum left ventricular pressure was also markedly reduced in the HS group (18.7 ± 12.1 mmHg) compared to control FS sample (52.2 ± 14.4 mmHg, *p* < 0.05).

Conversely, Figure 1c demonstrates that the minimum left ventricular pressure was substantially elevated in HS rats, averaging 48.8 ± 14.1 mmHg compared to only 17.2 ± 12.0 mmHg in the FS group (*p* < 0.05). This suggests an increased diastolic load in animals subjected to a high-salt diet.

**Figure 2:**
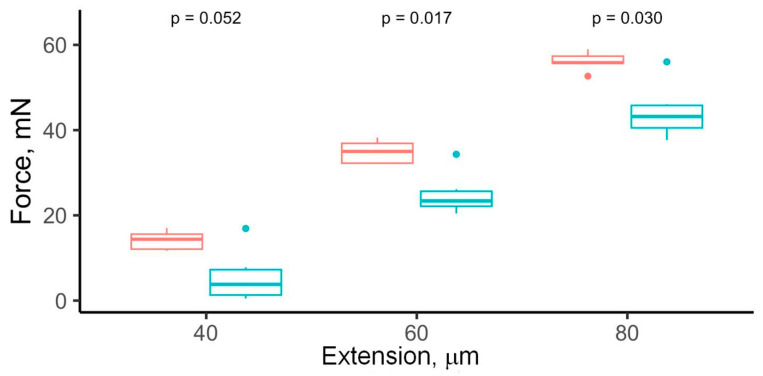
Stretch force applied for rat myocardium strip extension at 40 µm, 60 µm, and 80 µm extensions. Blue color indicates FS tissues, while red indicates HS tissues.

Figure 2 shows the force required to stretch the isolated myocardium strips of the same dimensions. At a 40 µm extension, the difference between the groups approached statistical significance (14.1 ± 2.3 mN vs. 5.6 ± 6.2 mN, *p* = 0.052), with the high-salt group demonstrating a greater stretch force compared to the low-salt group. As the extension increased to 60 and 80 µm, the differences became statistically significant (34.9 ± 2.8 mN vs. 24.9 ± 5.0 mN, *p* = 0.017; and 56.1 ± 2.3 mN vs. 44.4 ± 6.5 mN, *p* = 0.030, respectively). Thus, the myocardium from the high-salt group required a substantially greater force to achieve the same degree of deformation, indicating increased myocardial stiffness under high-salt conditions.

### 2.2. Raman Spectroscopy Measurements

**Figure 3:**
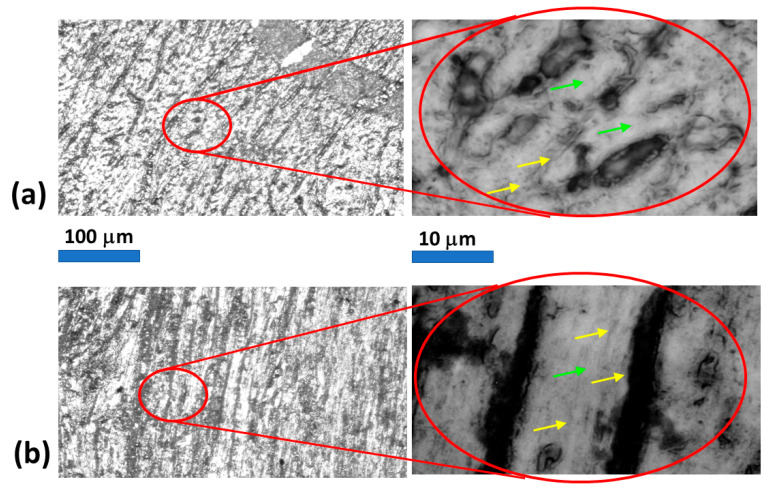
Representative microscopic images of unstained myocardial tissue sections. (**a**) FS sample; (**b**) HS sample. Yellow arrows mark cell–cell borders/interstitium, and green arrows mark intracellular spaces.

Raman spectra were acquired using an inVia Raman microscope–spectrometer with 785 nm excitation. Images of tissue sections were used to define several regions of interest (ROIs), allowing characterization of molecular structures in both the HS and FS groups. ROIs were selected to target either interstitial or intracellular spaces (see Figure 3).

**Figure 4:**
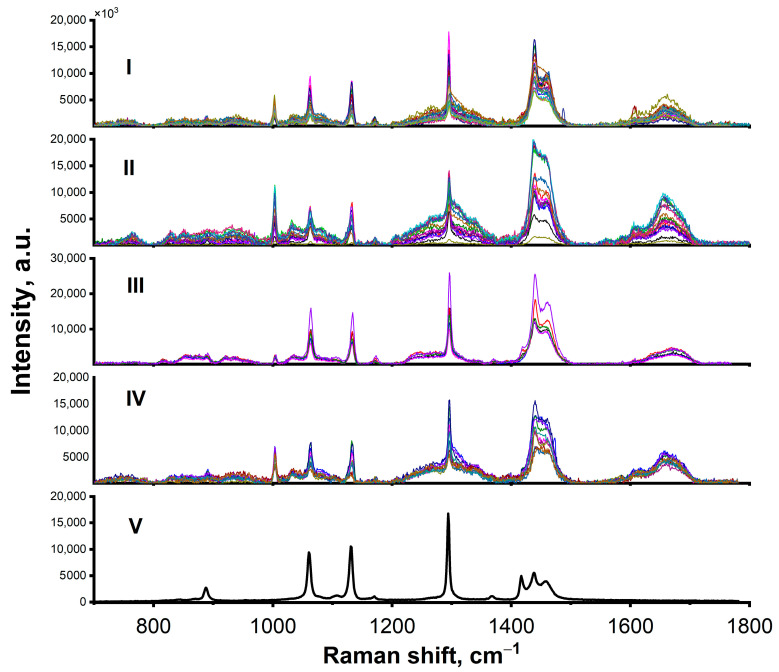
Representative Raman spectra measured in different regions of rat myocardial tissue. Control (FS) group: (**I**) spectra at cell boundaries/interstitium; (**II**) spectra from intracellular space. HS group: (**III**) spectra from cell boundaries/interstitium; (**IV**) spectra from intracellular space. Spectra are presented after background correction. (**V**) Reference paraffin spectrum, showing characteristic peaks in the range 1440–1460 cm^−1^, and at 1296, 1131, and 1062 cm^−1^.

Representative sets of Raman spectra obtained from interstitial and intracellular ROIs in the HS and FS myocardium are shown in Figure 4I–IV. In general, the spectra revealed characteristic Raman peaks of major tissue components [24] with spectral variations reproducibly detected within similar areas of each sample.

For reference, the Raman spectrum for paraffin is presented in Figure 4V. Characteristic paraffin peaks (in the range of 1440–1460 cm^−1^ and at 1296, 1131, and 1062 cm^−1^ [25]) were still detectable in some myocardial spectra, reflecting residual paraffin even after the deparaffinization procedure. These peaks appeared relatively intense compared to the spectral features of the biological samples.

**Figure 5:**
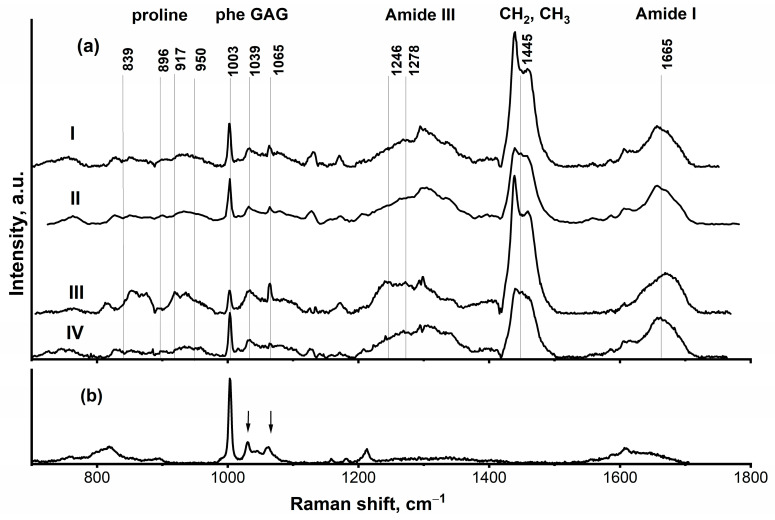
(**a**) Typical average spectra from different areas of the myocardial tissue samples. Control (FS diet) group: spectrum measured at the cell boundary/interstitium (I) and intracellular (II) space. High-salt (HS diet) group: spectrum measured at the cell boundary/interstitium (III) and intracellular (IV) space. (**b**) Spectrum of a GAG solution (sodium heparin) with marked (with arrows) peaks of sulphate groups (1039 and 1065 cm^−1^).

The most representative spectra from each morphological region of interest were averaged for further analysis, and the paraffin spectral contribution was subtracted, since its overlap with the tissue spectra complicated the analysis. The resulting spectra are shown in Figure 5a.

The region of 1030–1080 cm^−1^ is characteristic of glycosaminoglycan (GAG) vibrations. The spectra of various GAG-containing samples exhibit several common spectral features, most notably in the ranges of 1030–1070 cm^−1^ and around 1380 cm^−1^. Figure 5b presents the spectrum of a sodium heparin solution as an example of a GAG, revealing the peaks of the sulphate groups (1039 and 1065 cm^−1^) [26]. In the measured spectrum the peak at 1002 cm^−1^, is likely corresponding to the C–C stretching (phenyl-ring breathing) mode of a solvent, benzyl alcohol, a component of the sodium heparin solution used [27].

The range of 1220–1320 cm^−1^ includes the Amide III band. This band originates mostly from C–N stretching and N–H bending, with a smaller contribution from C–C stretching and C–H bending [28,29]. The spectral interval of 800–1100 cm^−1^ together with proteins backbone vibrations [29] specifically highlights the Raman signal of the amino acid proline [29,30], a major component of the collagen triple helix.

**Figure 6:**
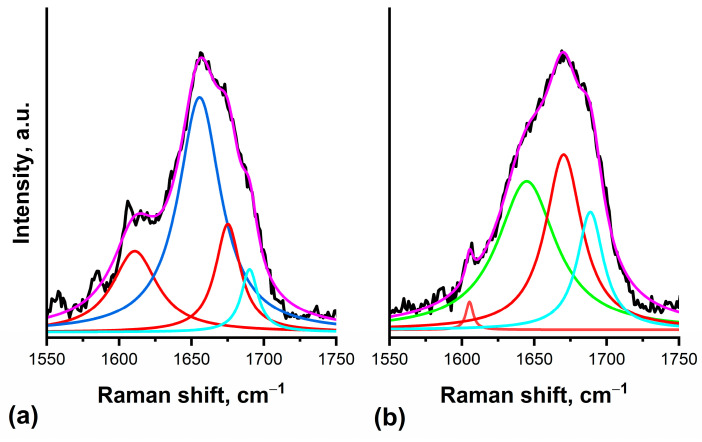
Lorentzian deconvolution of the Amide I band: (**a**) in the interstitial region of an FS sample (corresponding to spectrum I in Figure 5a); (**b**) in the interstitial region of an HS sample (corresponding to spectrum III in Figure 5a). Deconvolved peaks and their assignments are represented by colored curves as follows (see also Table 1): black, measured spectrum; red, β sheet; green, triple helix; blue, α helix; cyan, β-turn/random coil; purple, cumulative fitted curve.

The wide peak centered near 1665 cm^−1^ is the Amide I band, which arises mainly from vibrations of the C=O group of the polypeptide chain, as well as CN and NH groups [28,31]. Figure 6 shows the extended spectral region of the Amide I band. Figure 6a and Figure 6b present the results of our Lorentzian deconvolution of the Amide I peak in intracellular ROIs of the FS samples and interstitial areas of the HS samples, respectively; corresponding deconvolution parameters are given in Table 1.

## 3. Discussion

The results of relative measurements of hemodynamic and mechanical parameters indicate greater stiffness across all tested samples of the myocardium of animals exposed to the HS diet than the FS diet. This may reflect structural alterations in the myocardial tissue resulting from prolonged exposure to an HS diet. This fact has been discussed earlier in [16] in the studies of human heart stiffness. This study demonstrates that a high-salt (HS) diet induces significant myocardial stiffening and hemodynamic impairment, which we attribute to specific molecular-level alterations in the extracellular matrix (ECM). While the association between salt intake and cardiac fibrosis is known, our integrated biophysical approach reveals a more direct mechanism, sodium-driven disruption of the proteoglycan network, leading to maladaptive collagen remodeling.

Our Raman microspectroscopy data provide direct evidence that the paradigm of non-osmotic sodium storage in glycosaminoglycans (GAGs), previously established in skin and muscle [7], extends to the myocardium. The key finding is a pronounced difference between the interstitial regions of HS samples and all other tissue areas (Figure 5), particularly within the Amide I, Amide III, and 800–1100 cm^−1^ spectral ranges. This spatial specificity highlights the interstitial ECM as the primary site of sodium-induced pathology.

The most telling alterations were observed in the Amide I band, a key indicator of protein secondary structure. While the profiles were consistent in intracellular regions and control interstitium, the HS interstitium exhibited a marked shift. Deconvolution of the Amide I peak from spectra measured in interstitial areas of FS (Figure 6a) and HS (Figure 6b) samples revealed a substantial reduction in α-helical content, a dominant β-sheet peak at ~1670 cm^−1^, and a distinct new peak at ~1644 cm^−1^. While the ~1644 cm^−1^ peak can be assigned to β-sheets in some contexts [28], its co-occurrence with a peak at 1669 cm^−1^ identifies it as a collagen marker [33]. Calculations [32] further align it with the Raman signature of the triple helix [32,33], consistent with the experimentally observed shoulder at 1630–1640 cm^−1^ [33,34]. This interpretation is supported by a concurrent increase in proline-specific signals (800–1000 cm^−1^), decreasing relative signal of phenylalanine at 1003 cm^−1^—amino acid of low content in collagen triple helix, and an elevated *I*_1246_/*I*_1278_ ratio in the Amide III band [35], collectively indicating a qualitative shift towards a stiffer, proline-rich collagen phenotype [36]. This provides a direct molecular correlate for the observed increase in tissue rigidity.

The slight shifts observed in the deconvolved Amide I peaks for FS and HS samples can be attributed to alterations in the protein microenvironment and associated conformational changes, both linked to the pathological condition.

It should be emphasized that the purpose of the fitting of the Amide I region in the present study was not to provide an exhaustive molecular assignment of individual vibrational modes, but rather to enable a consistent comparative analysis between experimental groups under identical fitting constraints. The same fitting model and initial parameters were therefore applied uniformly to all spectra in order to minimize user-dependent bias and to allow robust relative comparisons.

It should also be noted that the fitted bands in the Amide I region may contain mixed contributions from different amino acid residues and protein secondary structure elements. Consequently, the peak assignments should be regarded as approximate and phenomenological. Absolute values of fitted peak positions and linewidths are subject to instrumental resolution and preprocessing limitations and are therefore interpreted with caution.

More complex deconvolution models of the Amide I envelope, involving a larger number of components, have been reported in the literature. However, such approaches typically require higher signal-to-noise ratios and larger datasets to avoid overfitting. In the present study, a simplified fitting strategy was intentionally chosen as a compromise between model complexity and robustness. Importantly, the conclusions of this work do not rely on the precise assignment of individual fitted peaks, but rather on consistent and reproducible trends observed between experimental groups.

We also observed evidence of GAG perturbation. The relative intensity of sulfate group vibrations in the 1030–1080 cm^−1^ range was significantly increased in the HS interstitium (Figure 5a, spectrum III). Specifically, the peaks at ~1066 cm^−1^ (2-O-sulfate) and 1030–1040 cm^−1^ (N-sulfate) were enhanced relative to the phenylalanine reference peak at 1003 cm^−1^. We posit that these spectral changes reflect the electrostatic binding of Na^+^ to these negatively charged groups [37], effectively “shielding” them. This charge shielding is not a passive event. The proteoglycan matrix is stabilized by a delicate balance of electrostatic and hydrogen-bonding interactions, including those between GAG sulfates and tyrosine residues in core proteins [7,37]. Sodium binding disrupts this balance, likely inducing conformational changes in GAG chains and, critically, severing their functional cross-links with collagen.

The exact positions of GAG peaks are influenced by both the specific GAG type and the local environment [38]. Therefore, the observed changes in Raman signal intensity within the corresponding spectral region can likely be attributed to the influence of Na^+^ ions, which presumably interact with the charged groups on the GAGs.

The observed collagen remodeling suggests a potential mechanistic link to the primary GAG alterations identified in our spectra. We therefore propose a plausible two-part model to interpret these findings. First, it is conceivable that the sodium-induced disruption of the GAG network could create a permissive biochemical and mechanical environment conducive to fibroblast-mediated collagen deposition. Second, the loss of physical tethering between GAGs and collagen might mechanically uncouple existing fibrils, potentially promoting the maladaptive deposition of a stiffer collagen variant. Together, this interpretation positions sodium as a potential molecular trigger for ECM dysregulation.

Furthermore, our findings can be contextualized by existing evidence showing that chronic high sodium exposure upregulates enzymes involved in GAG polymerization [7,38]. Thus, the intensified GAG Raman signal may reflect a dual effect: a conformational change in existing GAGs combined with a potential increase in GAG content. If correct, this synergistic effect could powerfully exacerbate interstitial stiffening. Additionally, the resulting GAG restructuring might promote local inflammation and increased tissue hydrophilicity [38], processes that could further modify tissue mechanics.

## 4. Materials and Methods

All animal experiments were conducted in accordance with the European Union Directive 2010/63/EU on the protection of animals used for scientific purposes and the Guide for the Care and Use of Laboratory Animals [39]. The study protocol was approved by the Ethics Committee of the Russian National Research Medical University prior to the initiation of the study.

### 4.1. Experimental Design and Animal Groups

A total of 15 male Wistar rats, aged 15–16 weeks, were randomly assigned to two dietary groups based on comparable initial body weights: a low-salt (free-of-salt, FS) group (*n* = 7; 297.4 ± 68.4 g) and a high-salt (HS) group (*n* = 8; 252.0 ± 67.4 g).

The animals were housed in standard laboratory cages under controlled environmental conditions (22 ± 2 °C, 12-h light/dark cycle, 50–60% humidity) with free access to food and deionized water. Beginning in the third week of the study, the rats were placed on their respective experimental diets. Each animal received 15 g of feed per 200 g of body weight daily. Both diets were based on standard rat chow (composition: 23% protein, 5% fat, 4% fiber, with balanced micronutrients) and were modified only in their sodium content. The FS diet contained 0.2 mEq of sodium per 200 g of body weight per day, while the HS diet provided 2.0 mEq of sodium per 200 g of body weight per day [40,41]. Concentrations of all other electrolytes (K^+^, Ca^2+^, Mg^2+^, Cl^−^) were identical in both diets to isolate the effect of sodium. Daily water intake was monitored by measuring changes in water bottle volume, and body weight was recorded weekly. The animals were maintained on their assigned diets for 8 weeks—a duration previously established to induce myocardial hypertrophy in rats [41].

### 4.2. Experimental Workflow

All animals from both dietary groups (*n* = 15 total; FS: *n* = 7, HS: *n* = 8) underwent the following sequential experimental procedures:

1. Langendorff perfusion for hemodynamic assessment;
2. Mechanical stiffness testing on freshly dissected tissue strips from the perfused hearts;
3. Histological preparation of tissue samples from the same hearts for subsequent Raman spectroscopic analysis.

This integrated approach enabled a comprehensive characterization of hemodynamic, mechanical, and molecular properties from the same hearts, thereby minimizing inter-animal variability and strengthening the correlation between the different levels of data.

### 4.3. Anesthesia and Heart Preparation

Upon completion of the 8-week feeding period, the animals were anesthetized via an intraperitoneal injection of methohexital (100 mg/kg body weight) and subsequently euthanized by decapitation. The hearts were rapidly excised and immediately processed for Langendorff perfusion.

### 4.4. Langendorff Perfusion and Hemodynamic Measurements

All isolated hearts (*n* = 15) were immediately cannulated via the aorta and perfused in the Langendorff mode using an ADInstruments Rodent Langendorff Apparatus PL3508B2-220 (ADInstruments Limited, Oxford, UK). Perfusion was conducted with a modified Krebs–Henseleit buffer containing the following (in mM): 118 NaCl, 4.7 KCl, 2.5 CaCl_2_, 1.2 MgSO_4_, 1.2 KH_2_PO_4_, 25 NaHCO_3_, and 11 glucose (pH 7.4). The buffer was continuously gassed with a mixture of 95% O_2_ and 5% CO_2_ and maintained at 37 °C. Coronary flow was maintained at a constant rate of 10 mL/min using a peristaltic pump (ML172 Minipuls 3, Gilson Incorporated, Middleton, WI, USA).

Hemodynamic monitoring was performed by inserting a fluid-filled latex balloon, connected to a physiological pressure transducer (MLT844, ADInstruments Ltd., Oxford, UK), into the left ventricle via the mitral valve. The balloon was inflated to set an end-diastolic pressure between 5 and 10 mmHg. Left ventricular pressure was recorded continuously at a sampling rate of 1000 Hz using an 8-channel data acquisition system (PL3508 PowerLab 8/35, ADInstruments Ltd., Oxford, UK). Following a 15-min stabilization period, 10 min of stable data were acquired for analysis. The derived hemodynamic parameters included: maximum left ventricular pressure, minimum left ventricular pressure, and the maximum rate of left ventricular pressure rise (*dp*/*dt*_max_). All data were analyzed using the LabChart Pro software, version 8.3.1 (ADInstruments Ltd., Oxford, UK).

### 4.5. Tissue Sampling and Processing

Following the completion of hemodynamic measurements (approximately 30 min total perfusion time), the hearts were immediately removed from the Langendorff apparatus and placed in ice-cold, oxygenated Krebs–Henseleit buffer. The left ventricle from each heart was carefully dissected and divided into two portions:

- Portion 1 (anterior and lateral walls): Immediately processed for mechanical stiffness measurements (see Section 4.6).
- Portion 2 (posterior wall and septal regions): Immediately fixed in 10% neutral-buffered formalin for histological preparation and subsequent Raman spectroscopy analysis (see Section 4.7).

This tissue allocation strategy ensured that both mechanical and spectroscopic analyses were performed on samples from the same hearts, enabling direct correlation between functional and molecular parameters.

### 4.6. Mechanical Stiffness Measurements

Myocardial tissue strips (approximately 2 × 1 × 1 mm^3^) were dissected from the anterior and lateral walls of the left ventricles. During preparation, the tissue was maintained at 4 °C in oxygenated Krebs–Henseleit buffer to preserve viability. Measurements were performed using a high-precision force transducer (HSE F30 Type 372, Hugo Sachs Elektronik–Harvard Apparatus GmbH, March, Germany) mounted on a programmable three-axis micromanipulator (MP-285, Sutter Instrument, Novato, CA, USA). Tissue strips were secured with 6-0 silk sutures, with one end attached to a stationary hook and the other to the force transducer. Prior to testing, samples were equilibrated for 30 min under a resting tension of 0.5 mN to establish the resting length (*L*_0_).

The objective was to determine the Young’s modulus of the passive rat myocardium. Passive mechanical properties were assessed by applying a controlled uniaxial stretch to three extension levels beyond *L*_0_: 40 µm, 60 µm, and 80 µm. At each extension, the force required to stretch the myocardium was recorded after a 2-min equilibration period. Young’s modulus was calculated from the stress–strain relationship at the 60 µm and 80 µm extensions. Stress (*σ*) was determined as the force divided by the cross-sectional area of the tissue strip, and strain (*ε*) was calculated as the extension divided by *L*_0_. The elastic modulus (*E*) was derived from the slope of the linear portion of the stress–strain curve (*E* = Δ*σ*/Δ*ε*). The primary focus was on identifying relative differences in myocardial stiffness between the dietary groups.

### 4.7. Sample Preparation for Raman Spectroscopy

For Raman spectroscopic analysis, myocardial tissue samples from the posterior wall and septal regions of the left ventricles were fixed in 10% neutral-buffered formalin for 24 h immediately following Langendorff perfusion. The fixed tissue blocks were then dehydrated through a graded ethanol series (70%, 80%, 95%, 100%; 1 h per concentration), cleared in xylene (two changes, 1 h each), and embedded in paraffin wax. Serial sections of 3 µm thickness were cut from each paraffin block (2–3 sections per animal, separated by 30 µm to avoid sampling the same tissue plane), mounted on glass slides, and air-dried. Subsequently, the sections were deparaffinized according to a standard FFPE (Formalin-Fixed Paraffin-Embedded) protocol by immersion in xylene (2 × 5 min) followed by rehydration through a graded ethanol series (100%, 95%, 70%, 50%; 3 min each) and a final rinse in distilled water.

### 4.8. Raman Spectroscopy

Raman spectra were acquired using a Renishaw inVia Raman Microscope spectrometer (Renishaw PLC, New Mills, UK) equipped with a 785 nm diode laser. The integrated confocal microscope was used for sample imaging. For both imaging and spectral acquisition, an NPlan 50×/0.50 objective with an 8-mm working distance (Leica Microsystems, Wetzlar, Germany) was employed. The typical acquisition time was 100 s per spectrum, with a spectral resolution of 1 cm^−1^. The laser power at the sample surface was maintained between 1.27 and 2.57 mW to prevent thermal and photochemical damage.

Characteristic regions of interest (ROIs) were visually identified from microscopic images of the tissue sections, allowing for the differentiation of interstitial areas from intracellular (cytoplasmic) regions. From each animal, 2–3 tissue sections were analyzed. Within each section, 4–6 ROIs were randomly selected from both interstitial and intracellular regions based on morphological criteria, resulting in approximately 8–18 ROIs per animal. In each ROI, 10–20 Raman spectra were collected and averaged to generate a representative spectrum for that specific region. In total, approximately 80–360 individual spectra per animal were acquired for comprehensive analysis.

Spectral pre-processing was performed using the WiRE 4.1 software (Renishaw PLC, New Mills, UK) and Origin 2018 (OriginLab Corp., Northampton, MA, USA). Post-processing included cosmic ray removal, baseline correction using polynomial fitting, normalization to the phenylalanine peak at 1003 cm^−1^, and background subtraction. Despite deparaffinization, residual paraffin peaks were occasionally observed; consequently, an averaged paraffin reference spectrum was subtracted from the tissue spectra after normalization to remove these contributions.

Spectral comparisons enabled the identification of features characteristic of interstitial and intracellular regions. Pure paraffin and a 10 mg/mL sodium heparin solution in benzyl alcohol were measured as reference standards under identical instrumental conditions.

### 4.9. Statistical Analysis

The data are presented as mean ± standard deviation (SD). Statistical analyses were performed using GraphPad Prism version 9.0 (GraphPad Software, San Diego, CA, USA), MATLAB R2021a (MathWorks, Natick, MA, USA), and the R statistical language (version 4.3.1; R Foundation for Statistical Computing, Vienna, Austria).

The normality of data distribution for hemodynamic and mechanical parameters was assessed using the Shapiro–Wilk test. Comparisons between the FS and HS groups were performed using unpaired, two-tailed Student’s *t*-tests for normally distributed data or Mann–Whitney *U* tests for non-normally distributed data. Statistical significance was defined as *p* < 0.05.

For all measurements, each heart provided one averaged value (for hemodynamic parameters and mechanical stiffness) or multiple regional values (for Raman spectral characteristics from different ROIs). The total *n* = 15 represents 15 independent animals—7 on the low-salt diet and 8 on the high-salt diet.

The sample size (*n* = 15 total) was determined based on preliminary data to detect an approximately 20% difference in myocardial stiffness with 80% power at *α* = 0.05.

## 5. Conclusions

The observed increase in myocardial stiffness, supported by distinct alterations in Raman spectra, provides compelling evidence that dietary sodium loading induces significant functional and structural modifications in the myocardial extracellular matrix, specifically within the glycosaminoglycan (GAG)–collagen complex. Our integrated biophysical approach demonstrates that a high-salt diet results in a stiffer myocardium, characterized molecularly by a shift in collagen conformation towards a proline-rich, triple-helical structure and an altered GAG environment indicative of sodium binding.

These findings align with previous reports that high salt intake promotes fibrosis and elevates TGF-β_1_ levels in cardiac and renal tissues of both normotensive and hypertensive rats [42]. Crucially, our study extends this understanding by proposing a direct biophysical mechanism: the electrostatic binding of Na^+^ to GAGs disrupts the proteoglycan network, which in turn triggers maladaptive collagen remodeling, ultimately increasing tissue rigidity. This sodium-induced structural remodeling provides a plausible mechanistic basis for the development of diastolic dysfunction, independent of or concurrent with hypertensive effects.

Future work should focus on temporal studies to delineate the sequence of these molecular events and explore therapeutic strategies aimed at preserving the integrity of the myocardial matrix in the context of high sodium exposure.

## Figures and Tables

**Table 1:** Peaks deconvolution parameters and assignments in Figure 6.

		Peak 1	Peak 2	Peak 3	Peak 4
FS sample (spectrum I)	Peak center, cm^−1^	1610.8 ± 0.9	1655.6 ± 0.95	1675.2 ± 0.8	1695.2 ± 0.8
	Peak width (FWHM), cm^−1^	37.6 ± 2.9	35.6 ± 2.4	21.0 ± 5.5	14.2 ± 3.1
HS sample (spectrum III)	Peak center, cm^−1^	1605.3 ± 0.5	1644.8 ± 1.7	1670.3 ± 0.7	1688.8 ± 0.8
	Peak width (FWHM), cm^−1^	6.0 ± 1.9	49.0 ± 3.4	30.8 ± 4.6	22.1 ± 2.4
	Peak assignment	β sheet [28]	α helix [28,31] triple helix [32] * collagen [33] *	β sheet [28,31] collagen [33]	β turn [28] random coil [31]

* Peak 2 assignment for Spectrum III only.

## Data Availability

The data that support the findings of this study are available from the corresponding author upon reasonable request.

## Abbreviations

The following abbreviations are used in this manuscript:

HS	“High-Salt”, related to increased salt intake
FS	“Free-Salt”, related to normal salt consumption
ROI	Region of interest
GAG	Glycosaminoglycan
FFPE	Formalin-Fixed Paraffin-Embedded
Phe	Phenylalanine
